# EVALUATION OF THE ANTEROLATERAL LIGAMENT OF THE KNEE IN MAGNETIC RESONANCE MRI: CASE SERIES

**DOI:** 10.1590/1413-785220233102e264848

**Published:** 2023-05-01

**Authors:** JOÃO PAULO FERNANDES GUERREIRO, AMANDA BREVILHERI BENASSI MANINI, DAVID BONINI VIEIRA CAMPANHÃ, GIOVANA ORTIZ ZENDRINI, PAULO ROBERTO BIGNARDI, MARCUS VINICIUS DANIELI

**Affiliations:** 1 Pontifícia Universidade Católica do Paraná, Faculdade de Medicina, Londrina, PR, Brazil.; 2 Hospital Evangélico de Londrina, Londrina, PR, Brazil.; 3 Hospital de Ortopedia Uniort.e, Londrina, PR, Brazil.

**Keywords:** Anterolateral Ligament, Anterior Cruciate Ligament, Ligaments Articular, Magnetic Resonance Imaging, Ligamento Anterolateral, Ligamento Cruzado Anterior, Ligamentos Articulares, Imageamento por Ressonância Magnética

## Abstract

**Objective:**

To evaluate the citation of the ligament in the magnetic resonance imaging (MRI) reports and confirm its presence and injury in the images of exams performed in the acute phase retrospectively.

**Methods:**

In total, 103 patients who underwent anterior cruciate ligament (ACL) reconstruction in 2019 were included. The images were reanalyzed by two radiologists.

**Results:**

In the first analysis, only one report mentioned the anterolateral ligament (ALL) and its injury (0.97%). On reanalysis, ALL was visualized in almost all cases (95% and 97%). An injury was found in 53 (51.5%) cases by radiologist A and in 56 (54.4%) cases by radiologist B. The injury was diagnosed by both in 39 (37.9%) cases (p < 0.0001). Radiologists disagreed regarding the injury (Kappa = 0.411).

**Conclusion:**

The reports failed to describe the ligament and diagnose a significant number of injuries. The analysis of conventional resonance images still presents divergences in the diagnosis of ALL injury associated with the ACL among radiologists. ***Level of Evidence IV, Case Series.***

## RESUMO

**Objetivo:**

Avaliar, de forma retrospectiva, a citação do ligamento anterolateral (LAL) em laudos de ressonância magnética (RM) e confirmar sua presença e lesão nas imagens de exames feitos na fase aguda.

**Métodos:**

Foram incluídos 103 pacientes submetidos à reconstrução do ligamento cruzado anterior (LCA) em 2019, cujas imagens foram reanalisadas por dois radiologistas.

**Resultados:**

Em primeira análise, apenas um laudo citava o LAL e sua lesão (0,97%); enquanto na reanálise, o LAL foi visualizado em quase todos os casos (95% pelo radiologista A e 97% pelo radiologista B). Foi encontrada lesão em 53 (51,5%) casos pelo radiologista A e 56 (54,4%) pelo radiologista B. Lesão foi diagnosticada por ambos em 39 (37,9%) casos (p < 0,0001). Houve divergência entre os radiologistas com relação à lesão (KappaL = 0,411).

**Conclusão:**

Os laudos deixaram de descrever o ligamento e diagnosticar um número significativo de lesões. A análise das imagens convencionais de ressonância ainda gera divergências no diagnóstico da lesão do LAL associada ao LCA entre os radiologistas. **Nível de Evidência IV, Série de Casos.**

## INTRODUCTION

Anterior cruciate ligament (ACL) injuries are among the most frequent injuries in orthopedics, affecting mainly active young people, which lead to reduced activity due to joint instability, with an incidence of 200,000 reconstructions per year in the USA.^1^ ^), (^ ^2^ In the past, reconstructions were exclusively extra-articular, becoming openly intra-articular, arthroscopically intra-articular. Today, extra-articular reinforcement associated with arthroscopic intra-articular reconstruction is discussed. ^(^ ^3^

The surgical method must be reconsidered due to the high rate of new ACL injury, which ranges from 6 to 28%, even with proper technical performance. ^(^ ^3^ Thus, the reinforcement or reconstruction of the anterolateral ligament (ALL) was proposed to cases of re-rupture. ^(^ ^4^ ^), (^ ^5^ The ALL is considered a distinct ligament structure in the third layer of the lateral compartment of the knee, being posterior and proximal to the lateral femoral epicondyle and with insertion in the anterolateral face of the tibia, halfway between the fibular head and Gerdy tubercle. ^(^ ^6^ Poor healing of the ALL injury occurs in 70% of patients undergoing isolated ACL reconstruction after one year. ^(^ ^5^ ^), (^ ^7^ Biomechanical studies show that in the combined injury of ACL and ALL, isolated ACL reconstruction does not reestablish normal knee biomechanics. ^(^ ^8^ In a clinical study, the combined ACL and ALL injuries were associated with significantly unfavorable results in isolated ACL reconstruction. ^(^ ^5^

Despite the improvement proven when undergoing combined surgery, the indications for extra-articular procedure are currently based on clinical parameters, such as pivot shift severity, patient activity level, and the surgeon’s personal experience. ^(^ ^9^ However, studies based on routine preoperative magnetic resonance imaging (MRI) show that MRI is highly sensitive, specific, and accurate for detecting ALL abnormality in adults. ^(^ ^10^ ^), (^ ^11^ In pediatric patients, this detection may be inaccurate due to knee size. ^(^ ^12^

Based on this evidence, the appropriate diagnosis of this injury becomes essential in the routine analysis of knee magnetic resonance imaging. Thus, this study questions the efficiency of conventional MRI in orthopedics for this evaluation. The hypothesis is that anterolateral ligament injuries are not being properly visualized and are not being reported by radiologists.

### Objectives

To evaluate the citation of the ligament in MRI reports, to evaluate the presence of the anterolateral ligament actively and retrospectively in MRI images by two different radiologists, and to evaluate the number of injuries associated with anterolateral ligament in cases subjected to surgery for ACL reconstruction.

## METHODS

This study was conducted after approval by the Research Ethics Committee (CEP) of the institution, according to opinion no. 4,811,548. This is an observational cross-sectional study. Patients of both sexes, over 18 years of age, operated for primary anterior cruciate ligament injury without correlation with associated ligament reconstruction and who underwent surgical treatment in 2019 were included; with preoperative MRI performed within one month after sprain with ACL injury (acute phase). Patients without MRI within one month after sprain with ACL injury (acute phase) were excluded.

In a single stage, two radiologists reanalyzed separately the MRI images, in search of visualization and injuries in the ALL. The definition of injury was previously described as changes in thickness, course, and/or edema around the ligament region. ^(^ ^10^ ^), (^ ^11^ No examination was considered poor quality or excluded.

For statistical analysis, the number of ALL in the reports was compared before the study to those reported by radiologists during the reanalysis. The diagnoses of ALL injury before and after reanalysis were compared, considering injury the case reported in the resonance report before the study and the cases of agreement between the two radiologists in the reanalysis. Agreement in the diagnosis between the two radiologists in the reanalysis was also compared by estimating the kappa coefficient with the Software SPSS 23.0 (IBM Corp., Armonk, NY, USA). Categorical data were evaluated using McNemar’s test with continuity correction to compare samples paired by GraphPad’s free web QuickCale.

## RESULTS

**Figure 1:**
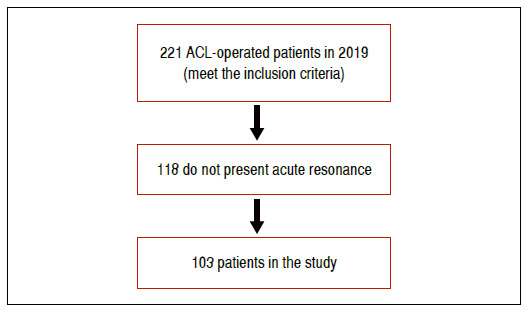
Study flowchart.

From the initial sample of 221 patients operated in 2019 by the group, 103 were included; 118 were excluded since MRI was not performed one month after sprain with ACL injury (Figure 1).

The participants were classified according to the citation of ALL in the report and its presence in images reanalyzed by radiologists.

Among all the reports analyzed, only one cited description and ALL injury. However, with the revaluation of the images by two radiologists in an active search for the ligament, this was visualized in 98 (95%) images by radiologist A and 100 (97%) images by radiologist B (Table 1). Among these, injury was found in 53 situations by radiologist A and in 56 by radiologist B (Table 2). They agreed in the diagnosis and considered a ligament injured in 39 cases (37.9%), one of which was already observed in the report made before the study (Table 3).

**Table 1:** ALL visualization.

	Previous analysis (N = 103)	Reanalysis - radiologist A (N = 103)	Reanalysis - radiologist B (N = 103)
**ALL visualization -N (%)**	1 (0.97)	98 (95)	100 (97)

**Table 2:** Diagnosis of ALL injury.

	Previous analysis (N = 103)	Reanalysis - radiologist A (N = 103)	Reanalysis - radiologist B (N = 103)	p-value
**Injury visualization in ALL -N (%)**	1 (0.97)	53 (51.5)	56 (54.4)	< 0.05

**Table 3:** Analysis of diagnoses in agreement among radiologists.

	Previous analysis (N = 103)	Reanalysis - radiologists (N = 103)	p-value
**Diagnosis of ALL injury -N (%)**	1 (0.97)	39 (37.9)	< 0.0001

Moreover, a significant divergence was found in the injury observation among radiologists. Among the 96 cases in which both radiologists identified the ligament on the images, only the kappa coefficient of 0.411 presented a moderate agreement between them (Table 4).

**Table 4:** Agreement in the diagnosis among radiologists.

	Value	Standardized asymptotic error^a^	Approximate T^b^	Approximate significance
**Kappa measure agreement**	0.411	0.093	4.026	0.000
**Number of valid cases**	96	-	-	-

^a^Not assuming the null hypothesis; ^b^Use of standard asymptotic error considering the null hypothesis.

## DISCUSSION

ALL is identified by MRI in 11-72% of cases, according to the sixth edition of Insall and Scott. ^(^ ^13^ On knees without injuries, Helito et al. ^(^ ^14^ identified the structure with magnetic resonance imaging of 1.5 Te in 81.8% of the cases in 2015. Another observational study on knees without injuries shows high sensitivity for ligament visualization and discusses the impasse of standardization of injury detection due to the difficulty in observing its entire extent due to the presence of accessory structures. ^(^ ^15^ Furthermore, studies present controversies about the location of frequent ligament abnormalities alongside the non-efficacy of the standard MRI sequence for such visualization, which also hinders diagnosis. ^(^ ^16^ When we evaluated knees with acute ACL injury, the ligament was present in 95-97% of radiologists’ evaluations only during reanalysis. In agreement with a retrospective comparative assay, ^(^ ^17^ the divergence in the visualization of anterolateral ligament injury among specialists in evaluations of images of the injured knee in the acute phase was also present in our study (Table 4). The concomitant ACL injury makes it difficult to observe the injury in ALL and suggests susceptibility to false results - positive and negative. ^(^ ^17^

Regarding the injury, both radiologists agreed on its diagnosis in 39 cases (37.9%). One of the first publications on the subject shows 32.6% of associated injuries in MRI images. ^(^ ^16^ More recently, three studies show rates of associated injuries close to 90%.^10^ ^), (^ ^11^ ^), (^ ^18^ In one of them, three-dimensional MRI images were used(3D), ^(^ ^18^ in the other, MRI images of the contralateral knee without injury were used as a comparison standard, ^(^ ^10^ and in the last, the evaluations were made by three scholars of the subject always using MRI of 1.5 Tesla. ^(^ ^11^ The evaluation with 3D MRI and the use of a contralateral knee resonance examination as a reference were scientifically effective, but difficult to apply during usual clinical practice. However, the most careful and rigorous evaluation, as in the last example, seems to be more feasible in daily clinical practice considering the variability of the ligament aspect among individuals and the presence of accessory structures. ^(^ ^16^

Despite several publications on the subject in the past decade worldwide and several publications by Brazilian authors on the subject^19^ in this series of cases, we found only one report (0.97%) containing information on ALL. Thus, we understand that it would be appropriate to increase the active search for ALL during the evaluation of images by radiologists in orthopedics, enabling other data for the surgeon to define if the associated reconstruction between the ACL and ALL in the acute phase will be made and facilitate the authorization of the procedures and materials necessary by healthcare insurers. Today, radiologists consider it essential to report the presence of LAL and changes in thickness, course, and edema around, when present, showing the probable injury. ^(^ ^10^ ^), (^ ^11^

This study presented some limitations and biases. First, the retrospective analysis of images were performed in several radiology services with different resonance devices and with resolutions ranging from 0.5 to 1.5 Tesla. Second, the series of cases encompassed only a single medical center. Finally, this study included a large number of patients operated with ACL injury, but many had not undergone acute phase imaging and, therefore, were excluded from the analysis.

## CONCLUSIONS

In this series of cases, we show that the reports no longer describe the ALL and diagnose a significant number of injuries. The analysis of conventional resonance images still presents divergences in the diagnosis of ALL injury associated with ACL among radiologists.
